# Outcomes of periodontal therapy in rheumatoid arthritis: The OPERA feasibility randomized trial

**DOI:** 10.1111/jcpe.13756

**Published:** 2022-12-16

**Authors:** Paola de Pablo, Stefan Serban, Isabel Lopez‐Oliva, Joanna Rooney, Kirsty Hill, Karim Raza, Andrew Filer, Iain Chapple, Thomas Dietrich

**Affiliations:** ^1^ Rheumatology Research Group Institute of Inflammation and Ageing, College of Medical and Dental Sciences, University of Birmingham Birmingham UK; ^2^ Department of Rheumatology Sandwell & West Birmingham NHS Trust Birmingham UK; ^3^ Department of Rheumatology University Hospital Birmingham NHS Foundation Trust Birmingham UK; ^4^ Periodontal Research Group, School of Dentistry, Institute of Clinical Sciences, University of Birmingham, and Birmingham Dental Hospital (Birmingham Community Healthcare Trust) Birmingham UK; ^5^ Department of Dental Public Health School of Dentistry, University of Leeds Leeds UK; ^6^ Department of Periodontology Institute of Dentistry, Barts and The London School of Medicine and Dentistry, Queen Mary University of London London UK

**Keywords:** clinical trial, epidemiology, general health, periodontal diseases, therapy

## Abstract

**Aim:**

Periodontitis is independently associated with rheumatoid arthritis (RA); however, there is limited data on whether periodontal treatment improves overall RA disease activity. We conducted a pilot feasibility randomized controlled clinical trial to test whether intensive periodontal therapy reduces RA disease activity in patients with active RA and periodontitis.

**Materials and Methods:**

The following inclusion criteria were applied: patients with RA and periodontitis, aged 18+, stable on treatment with disease‐modifying anti‐rheumatic drugs for ≥3 months, disease activity score (DAS28) ≥3.2, and DAS28 >5.1 only if patient unwilling to take biologics. Participants meeting the inclusion criteria were randomized to immediate intensive periodontal therapy or to delayed therapy (control group) administered by a dental hygienist in a secondary care setting. Data were collected at baseline and at 3 and 6 months of follow‐up. Participants randomized to the control group (delayed therapy) received the standard of care for the duration of the trial, including oral hygiene instructions delivered by a dental hygienist, and the same periodontal therapy as the intervention group after study completion (i.e., 6 months after randomization). The periodontal inflammation surface area was calculated using clinical attachment loss (CAL), periodontal probing pocket depth, and bleeding on probing. Cumulative probing depth was also measured. We examined the effect of periodontal therapy on periodontal outcomes and on clinical markers of disease activity in RA, as measured by the DAS28‐C‐reactive protein score as well as musculo‐skeletal ultrasound grey scale and power Doppler scores.

**Results:**

A total of 649 patients with RA were invited to participate in the study. Of these, 296 (46%) consented to participate in the screening visit. A sample of 201 patients was assessed for eligibility, of whom 41 (20%) did not meet the RA inclusion criteria and 100 (50%) did not meet the periodontal disease criteria. Among the 60 (30%) eligible participants, 30 were randomized to immediate periodontal therapy and 30 were allocated to the control group. The loss to follow‐up was 18% at the end of the trial. There were no major differences with regard to baseline characteristics between the groups. Periodontal therapy was associated with reduced periodontal inflamed surface area, cumulative probing depths, RA disease activity scores, and ultrasound scores over the course of the trial. There was no change in CAL.

**Conclusions:**

Overall, the trial was feasible and acceptable to the study participants. Recruitment to and satisfactory retention in a randomized controlled trial on the effect of periodontal treatment on RA patients is possible, albeit challenging. In this feasibility study of patients with RA and periodontitis, periodontal treatment resulted in significant improvements in periodontal disease outcomes and overall RA disease activity, although complete resolution of periodontal inflammation was difficult to achieve in some cases.

**Clinical Relevance**

*Scientific Rationale for Study*: There are limited data on whether periodontal treatment improves RA disease activity. We conducted a feasibility randomised controlled trial (RCT) testing whether intensive periodontal therapy reduces disease activity in patients with active RA and periodontitis.

*Principal Findings*: We successfully recruited and randomised patients with RA and periodontitis, with retention rates >80% over the trial. Complete resolution of periodontal inflammation was difficult to achieve in some cases.

*Practical Implications*: Recruitment and retention into a RCT on the effect of periodontal therapy on RA patients is possible. Apparent improvements in RA outcomes in this feasibility study suggest that a definitive trial is worthwhile.

## 1 INTRODUCTION

Rheumatoid arthritis (RA) is a common, chronic, immune‐mediated, inflammatory disease characterized by inflammation in the joints, ultimately leading to joint destruction and consequently functional impairment and disability (Smolen et al., 2016). In addition, patients with RA are more likely to develop cardiovascular diseases such as heart disease and stroke and are therefore at an increased risk of premature death. Several studies indicate that periodontitis (Hajishengallis, 2015), a common inflammatory disease of the gums surrounding the teeth and triggered by bacteria in the mouth, is associated with RA and can initiate and worsen inflammation in RA (de Pablo et al., 2008; Lundberg et al., 2008; de Pablo et al., 2009; Chen et al., 2013; de Pablo et al., 2014; Mikuls et al., 2014; Johansson et al., 2016; Kharlamova et al., 2016; Kindstedt et al., 2018; Lopez‐Oliva et al., 2018; Lopez‐Oliva et al., 2019a, 2019b; Gomez‐Banuelos et al., 2020; Albrecht et al., 2021; Hajishengallis & Chavakis, 2021). A limited number of small clinical studies in patients with RA have indicated that periodontal therapy aimed at reducing periodontal inflammation and the associated microbial burden can reduce systemic inflammation in patients with RA (summarized in Sun et al., 2021). If effective, periodontal therapy in RA patients who also suffer from chronic periodontitis would constitute a non‐pharmacological therapy for RA patients, which could be added to existing disease‐modifying anti‐rheumatic drug (DMARD) therapy. However, there is no data from pivotal randomized clinical trials (RCTs) on whether periodontal treatment improves overall RA disease activity. We conducted a pilot feasibility randomized trial with parallel arms to evaluate the efficacy of intensive periodontal therapy in reducing RA disease activity in patients with RA and periodontitis. The main objective of this study was to assess the feasibility of the proposed study protocol, patient recruitment, and retention to gauge the acceptability of the intervention and study procedures to patients and to gather pilot data on the effect size and variability of the effect of the intervention.

## 2 PATIENTS AND METHODS

### 2.1 Study sample

We recruited adults with RA according to established classification criteria for RA (Arnett et al., 1988; Aletaha et al., 2010) from outpatient rheumatology clinics in Birmingham (i.e. University Hospital Birmingham [UHB] and Heart of England [HEFT] NHS Foundation Trusts, and Sandwell & West Birmingham [SWB] NHS Trust). Following written informed consent, patients were invited to participate in a separate screening visit at the Birmingham Dental Hospital (BDH) to assess the following eligibility criteria for the trial:

#### 2.1.1 Inclusion criteria

Adults (18+ years) with RA on stable treatment with DMARDs for ≥3 months and with a disease activity score (DAS28) ≥3.2 or a DAS28 score >5.1 only if unwilling to take biologics, and meeting criteria for generalized Stage II–IV periodontitis, defined as clinical attachment loss (CAL) ≥4 mm on at least two non‐adjacent teeth and cumulative probing depth ≥40 mm (Dietrich et al., 2008; Papapanou et al., 2018).

#### 2.1.2 Exclusion criteria

Patients were excluded if they had a history of any of the following:

- Inflammatory rheumatic disease other than RA
- Any surgical procedure within 3 months prior to baseline
- Intra‐articular or parenteral glucocorticoids within 4 weeks prior to baseline
- Periodontal treatment within 12 months prior to baseline
- Significant concomitant disease, which would preclude study participation in the investigators' opinion
- Any dental condition that would preclude study participation in the investigators' opinion

### 2.2 Study implementation

#### 2.2.1 Randomization

Study participants were randomized to either immediate intensive periodontal therapy and maintenance administered by a dental hygienist in a secondary care setting (intervention group), or delayed treatment (control group). We used stratified randomization based on age, sex, and anti‐citrullinated protein auto‐antibodies (ACPA) status. Telephone randomization implemented by the Birmingham Clinical Trials Unit (BCTU) determined the treatment group allocation.

#### 2.2.2 Intervention group

A research‐trained dental hygienist (J.R.), blinded to RA disease activity measures and ultrasound findings, carried out all the intervention and education sessions at BDH. The study hygienist was a very experienced clinician, with many years of experience in performing non‐surgical periodontal therapy routinely. The intervention consisted of non‐surgical periodontal therapy consisting of sub‐gingival professional mechanical plaque removal (PMPR), which was performed under local anaesthesia using ultrasonic scalers and hand instruments, in addition to coaching in oral hygiene. The dental hygienist completed the intervention in 2–3 sessions. All sites with probing depth (PD) ≥4 mm and bleeding on probing (BOP) at the 3‐ or 6‐months follow‐up visits were re‐instrumented.

#### 2.2.3 Control group (delayed periodontal therapy)

Patients in the control group received an oral hygiene education session administered by the study dental hygienist at baseline. Patients were reviewed at 3 months and rescue treatment was planned for sites exhibiting CAL of more than 2 mm since baseline. After study completion at 6 months, patients received periodontal therapy outside the trial.

#### 2.2.4 RA management

The treatment of RA was in line with the national clinical recommendations at the time of the study. We added the periodontal treatment to the existing background treatment in participants randomized to the intervention arm. RA treatment modifications were permitted by each participant's GP or rheumatologist.

### 2.3 Data collection

We collected data on clinical markers of disease activity in RA, including ultrasound grey scale (USGS) and power Doppler scores, at baseline and at 3 and 6 months of follow‐up.

#### 2.3.1 RA disease activity

The same clinical examiner who was trained and calibrated by a rheumatologist (P.d.P.) and was blinded to periodontal treatment allocation collected disease activity measures, including tender joint count (TJC), swollen joint count (SJC), patient global visual analogue scale (VAS), and inflammation biomarkers (erythrocyte sedimentation rate [ESR] and C‐reactive protein [CRP]) to compute the disease activity score based on 28 joints (DAS28) (Prevoo et al., 1995). We report the results for DAS28‐CRP.

#### 2.3.2 Musculoskeletal ultrasound

We obtained USGS and power Doppler scores from metacarpo‐phalangeal (MCP) joints 1–5 and dorsal wrists (worst case grading of radio‐carpal, inter‐carpal, or ulnar‐carpal recesses) on both sides. Musculoskeletal ultrasound (MSUS) scans were performed prior to the dental examination by the same examiner (S.S.) who was trained and calibrated by a musculo‐skeletal ultrasonographer (A.F.) and was blinded to periodontal treatment allocation. The presence of USGS synovial hypertrophy and ultrasound power Doppler (USPD) enhancement were graded on a 0–3 scale, as previously described (Filer et al., 2011).

#### 2.3.3 Periodontal measures

All clinical periodontal assessments were carried out by the same dental surgeon throughout the study (ILO). For inclusion, volunteers needed to exhibit CAL ≥4 mm on at least two non‐adjacent teeth and cumulative PD ≥40 mm. For participants enrolled in the trial, periodontal examination was implemented at each study visit to monitor the periodontal response to therapy and to collect measures routinely used in practice including plaque and gingival indices, full‐mouth probing for periodontal probing depth (PPD), BOP, and CAL at six sites per tooth. We calculated the periodontal inflamed surface area (PISA) using CAL, PPD, and BOP. PISA is the surface area of the bleeding pocket epithelium in square millimetres, that is, it is a quantitative measure of the amount of inflamed periodontal tissue accounting for pocket probing depths (PPD) and number and size of affected teeth (Nesse et al., 2008).

#### 2.3.4 Biological measurements

We obtained non‐fasting blood samples at every study visit and measured the ESR levels locally and CRP levels at a central laboratory. We also collected oral samples, including sub‐gingival dental plaque, gingival crevicular fluid, and saliva (Lopez‐Oliva et al., 2018).

#### 2.3.5 Sample size and statistical methods

The targeted sample size was 60 randomized participants. This feasibility study also needed to establish that the periodontal treatment as delivered in this study achieved a minimum standard in terms of periodontal healing (PPD reduction at 3 and 6 months). A priori, we specified a standardized effect size of 1 for reduction of the mean PD as a reasonable minimum threshold criterion. The feasibility study had >90% power to detect such an effect size, even allowing for 20% loss to follow‐up.

Our primary aims were to assess the feasibility of an RCT of the efficacy of intensive periodontal therapy to reduce RA disease activity in patients with RA, to assess patient recruitment and retention, to gauge the acceptability of the intervention and study procedures to patients, and to gather pilot data on the effect size and variability of the effect of the intervention.

We used descriptive statistics to establish the proportion of patients with RA who were willing to participate in the study and who satisfied the inclusion criteria.

We used qualitative methods to gauge the acceptability of the study procedures and intervention as well as reasons for non‐participation (reported in Serban et al., 2019).

For randomized patients, we a priori specified a target minimum retention rate of 80% at 6 months.

Our secondary objective was to collect pilot data regarding the efficacy and safety of subgingival PMPR to reduce periodontal parameters and disease activity for patients with RA and periodontitis. No adjunctive antimicrobials were employed by design. We calculated summary statistics as appropriate. Descriptive results are reported as mean (SD), unless otherwise stated.

#### 2.3.6 Study oversight

The OPERA trial protocol was approved by the National Research Ethics Service (NRES) Committee West Midlands—South Birmingham Research Ethics Committees (REC Number 11/WM/0235, protocol number RG_10‐138) and registered via the Integrated Research Application System (IRAS). All study participants provided written informed consent.

The clinical trial was funded by the National Institute for Health Research (NIHR) Research for Patient Benefit (RfPB) programme (Reference number PB‐PG‐0609‐19100) and registered at the ISRCTN Register (registration number ISRCTN52833273; www.controlled-trials.com).

## 3 RESULTS

### 3.1 Recruitment and retention

A total of 649 patients with RA were invited to participate in the study between January 2014 and December 2016. Of these, 353 (54%) declined the invitation for a variety of reasons, including unwillingness to participate in trials (33%), other health priorities and comorbidities (24%), complete tooth loss (11%), self‐reported good dental health (4%), phobia of dentists (1%), lack of time due to work commitments (2.6%), language barriers (2.3%), and other undisclosed reasons (22%).

**FIGURE 1:**
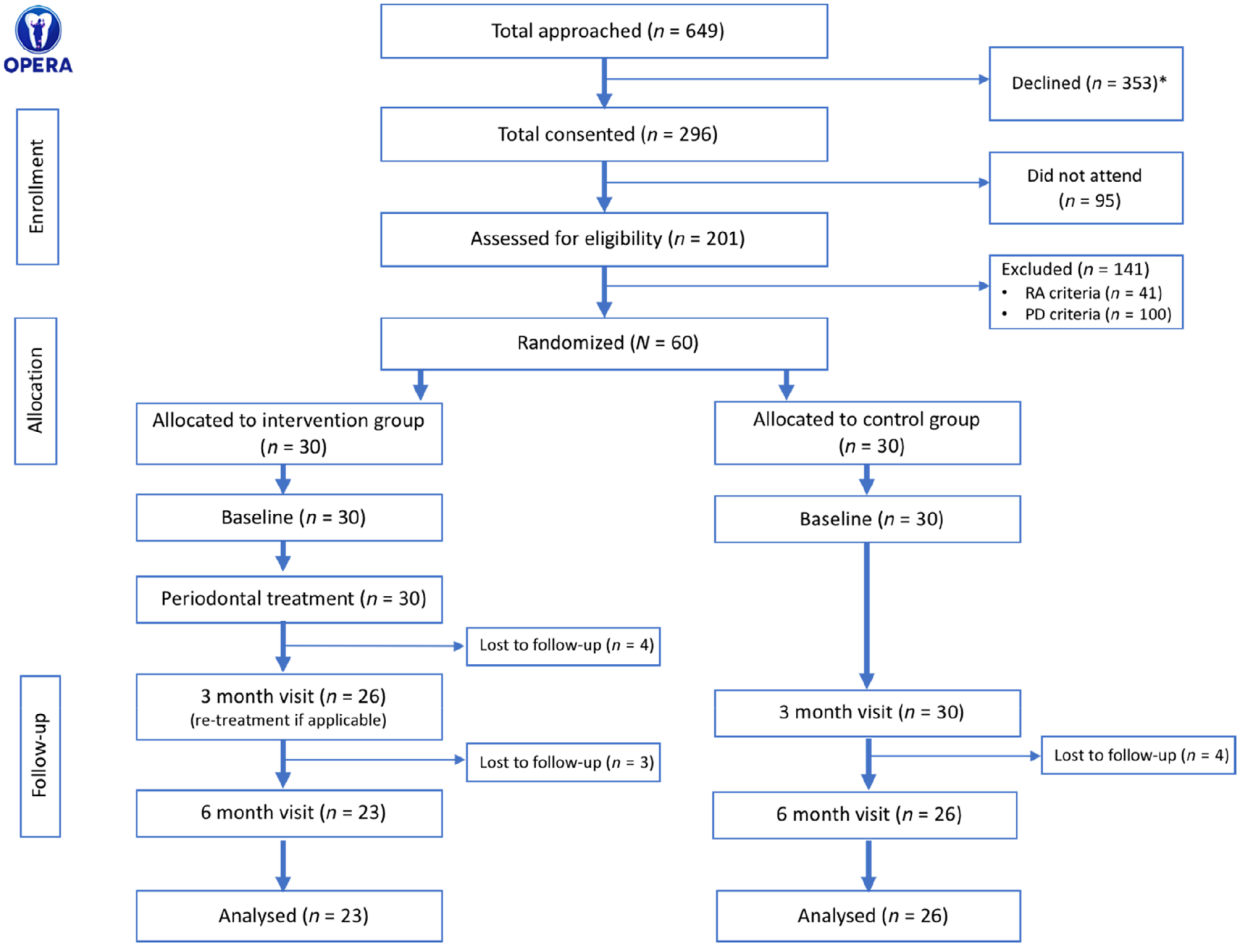
OPERA trial flow diagram. *A total of 353 (54%) patients with rheumatoid arthritis (RA) declined the invitation to participate in the study for a variety of reasons, including unwillingness to participate in trials (33%), other health priorities and comorbidities (24%), complete tooth loss (11%), self‐reported good dental health (4%), phobia of dentists (1%), lack of time due to work commitments (2.6%), language barriers (2.3%), and for undisclosed reasons (22%). PD, periodontal disease.

A total of 296 RA patients consented to participate in the screening visit at the dental hospital, of whom 95 (32%) did not attend. We assessed 201 individuals for eligibility. Of these, 41 (20%) did not meet the RA inclusion criteria and 100 (50%) did not meet the periodontal disease criteria. A total of 60 out of 201 (30%) participants met the eligibility criteria and were randomized to the intervention group (immediate periodontal treatment) or the control group (delayed periodontal treatment). The CONSORT (Schulz, Altman, Moher, & CONSORT Group, 2010) trial flow diagram is presented in Figure 1.

Of the total number of patients with RA initially approached, 31% attended the screening visit and 9.2% met the eligibility criteria. Study participants frequently cancelled or re‐booked appointments throughout the trial, making it difficult to adhere to a strict timeline for follow‐up visits. The overall loss to follow‐up was 18% at the end of the trial. There were more losses in the intervention group than in the control group at the 6‐month visit (23% vs. 13%, respectively).

### 3.2 Periodontal status

Overall, periodontal health characteristics at baseline were well balanced between the groups (Table 1). The number of sites with PPD ≥6 mm and PISA was slightly higher in the control group.

**TABLE 1:** Baseline characteristics

	Intervention group (immediate treatment)	Control group (delayed treatment)
*n*	30	30
Age, years	59	57
Female subjects, %	67	83
Race, %		
White	69	57
South‐Asian	19	33
Afro‐Caribbean	6	10
Other	6	0
Smoking, %		
Never smoker	37	54
Former smoker	37	23
Current smoker	26	23
No. of cigarettes, median (IQR)	10 (6–12)	15 (10–30)
BMI, mean (SD)	31 (8)	29 (7)
Systolic BP, mean (SD)	142 (21)	135 (23)
Diastolic BP, mean (SD)	87 (15)	85 (13)
Oral health characteristics		
Number of teeth, median (IQR)	22 (15–25)	25 (20–26)
Cumulative probing depth, median (IQR)	64 (44–80)	64 (56–90)
Mean probing depth (mm), mean (SD)	3.4 (0.7)	2.6 (0.6)
Bleeding on probing, %	30 (22)	17 (18)
Sites with PPD ≥6 mm, median (IQR)	5.1 (1, 8)	7 (1–9)
Periodontal inflamed surface (mm^2^), median (IQR)	374 (180–745)	390 (203–720)
RA disease activity characteristics		
CRP (mg/l), mean (SD)	3.75 (0.71–8.82)	3.73 (0.66–11)
ESR (mm/h), mean (SD)	18 (14)	26 (18)
Tender joint count (0–28), mean (SD)	12 (8)	13 (8)
Swollen joint count (0–28), mean (SD)	2 (2.8)	3 (3.6)
Patient's global assessment (0–100), mean (SD)	56 (23)	65 (18)
DAS28, mean (SD)	4.98 (1.17)	5.32 (0.99)
Ultrasound global scores, mean (SD)		
Ultrasound grey scale score, mean (SD)	10 (6.8)	12.1 (9.7)
Ultrasound power Doppler, mean (SD)	5.2 (5.6)	7.2 (8.4)
ACPA positive, %	76	89
Medications, %		
DMARDs	37	57
Biologics	33	33
NSAIDs	10	20
Steroids	20	33

Abbreviations: ACPA, anti‐cyclic citrullinated peptide antibodies; BMI, body mass index; CRP, C‐reactive protein; DAS28, 28‐joint disease activity score (calculated using ESR); DMARDs, disease‐modifying anti‐rheumatic drugs; ESR, erythrocyte sedimentation rate; IQR, inter‐quartile range; NSAIDs, non‐steroidal anti‐inflammatory drugs; RA, rheumatoid arthritis; SD, standard deviation.

**FIGURE 2:**
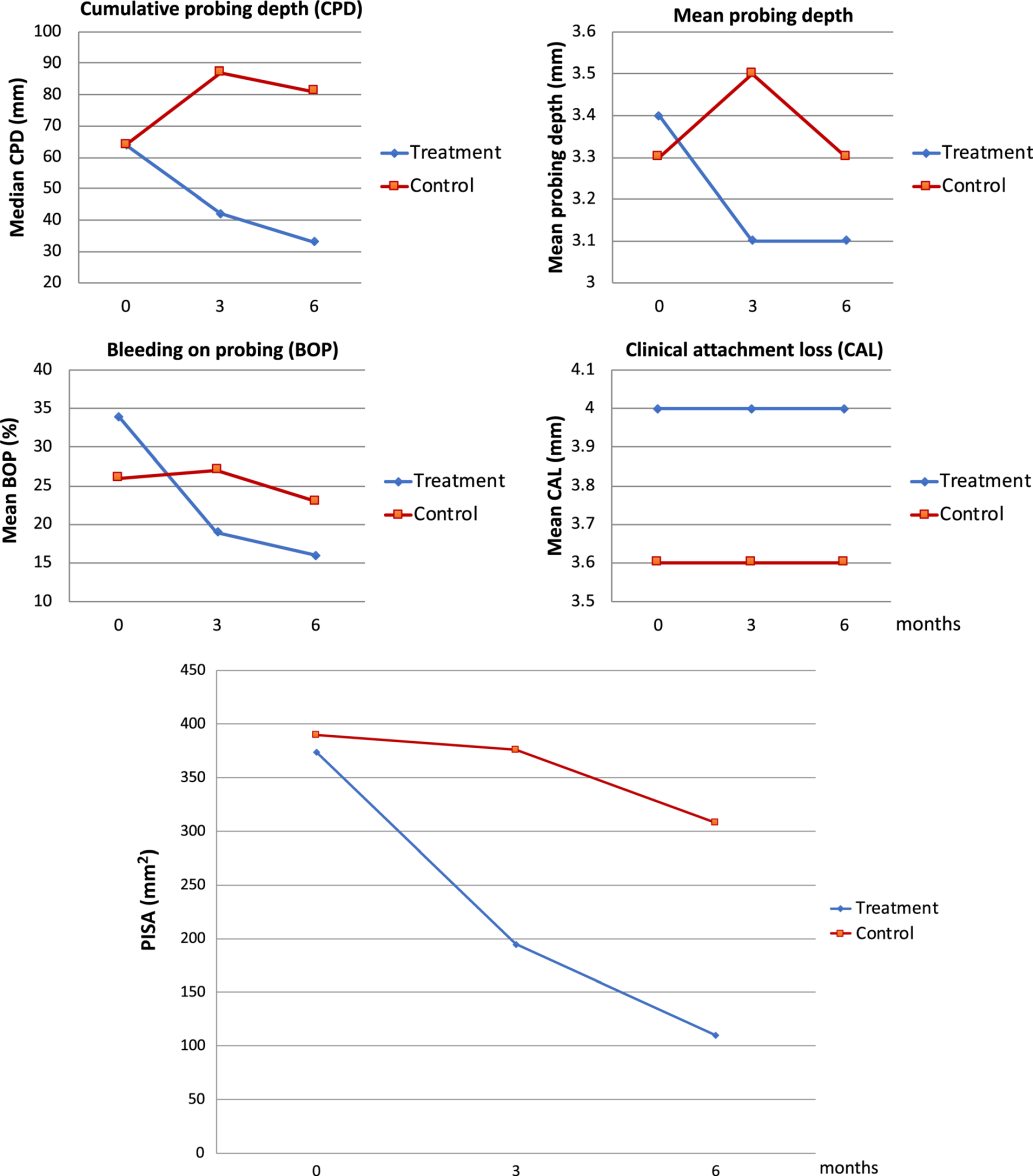
Periodontal disease measures. Periodontal inflammation surface area (PISA).

Except for CAL, all periodontal measures tended to improve after periodontal treatment in the intervention group compared with the comparator group over the course of the trial, including cumulative probing pocket depth (CPD), mean PPD, and BOP. Complete resolution of periodontal inflammation was not achieved, but there was a positive difference between groups at both 3 and 6 months in terms of cumulative PPD and PISA (Figure 2).

### 3.3 RA status

There were no major differences in the baseline RA disease activity characteristics between the groups (Table 1). Compared with the intervention arm, the control group had more individuals with a DAS28 >5.1 (41% vs. 57%, respectively). There were more smokers in the intervention group; however, the comparator group smokers tended to smoke more cigarettes than those in the intervention group. Participants in the control group tended to have slightly higher RA disease activity measures (except for CRP levels) and receive more conventional synthetic DMARDs (csDMARDS), steroids, and NSAIDs, but similar proportion of biologic DMARDs (bDMARDs) than those in the intervention group.

**FIGURE 3:**
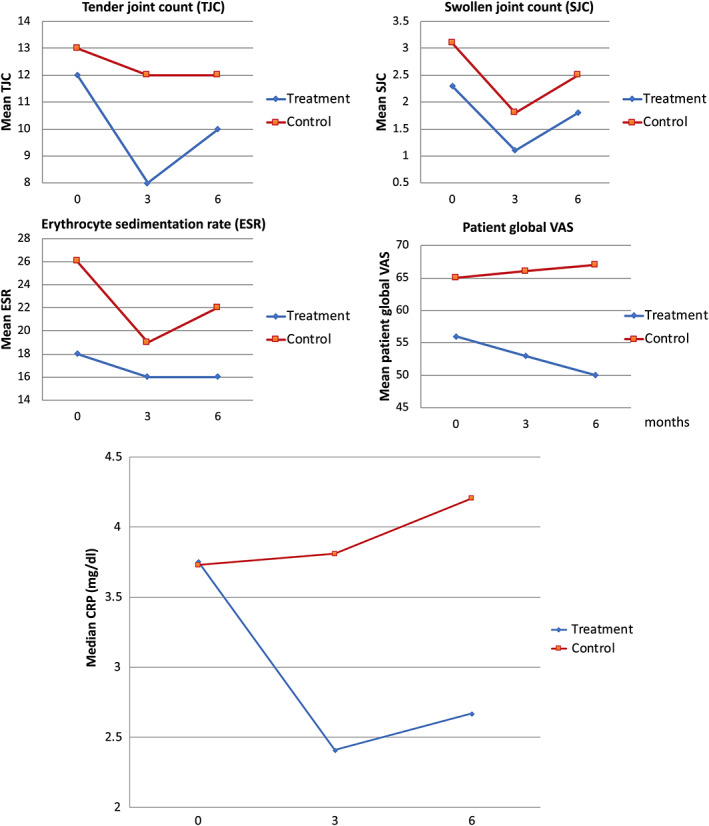
Rheumatoid arthritis disease activity measures

**FIGURE 4:**
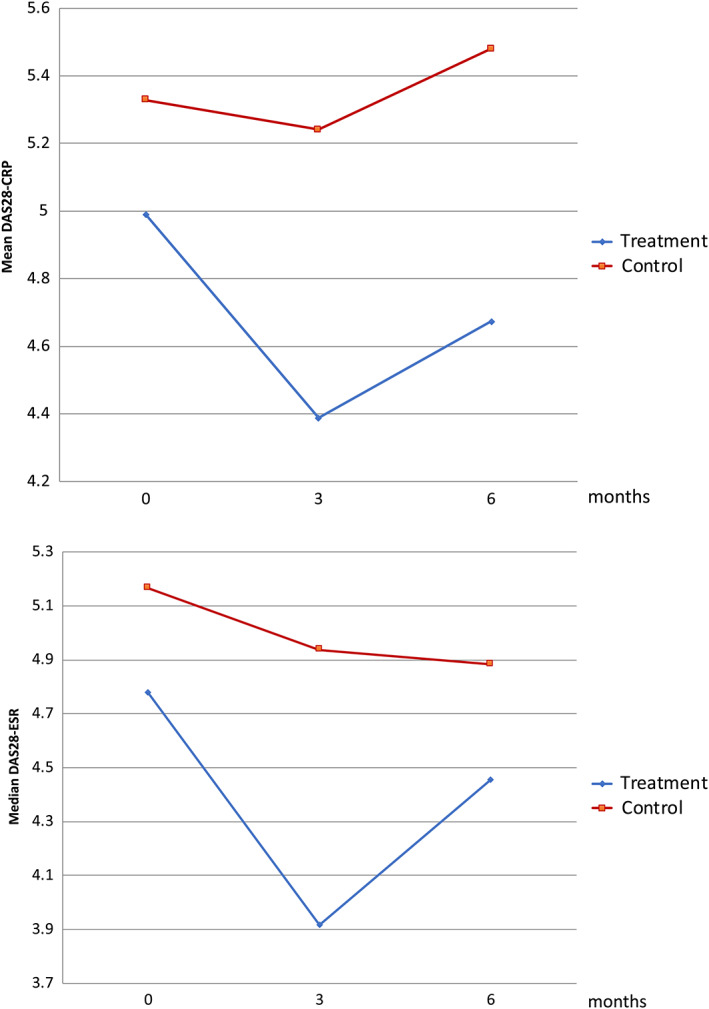
Rheumatoid arthritis disease activity score (DAS28)

**FIGURE 5:**
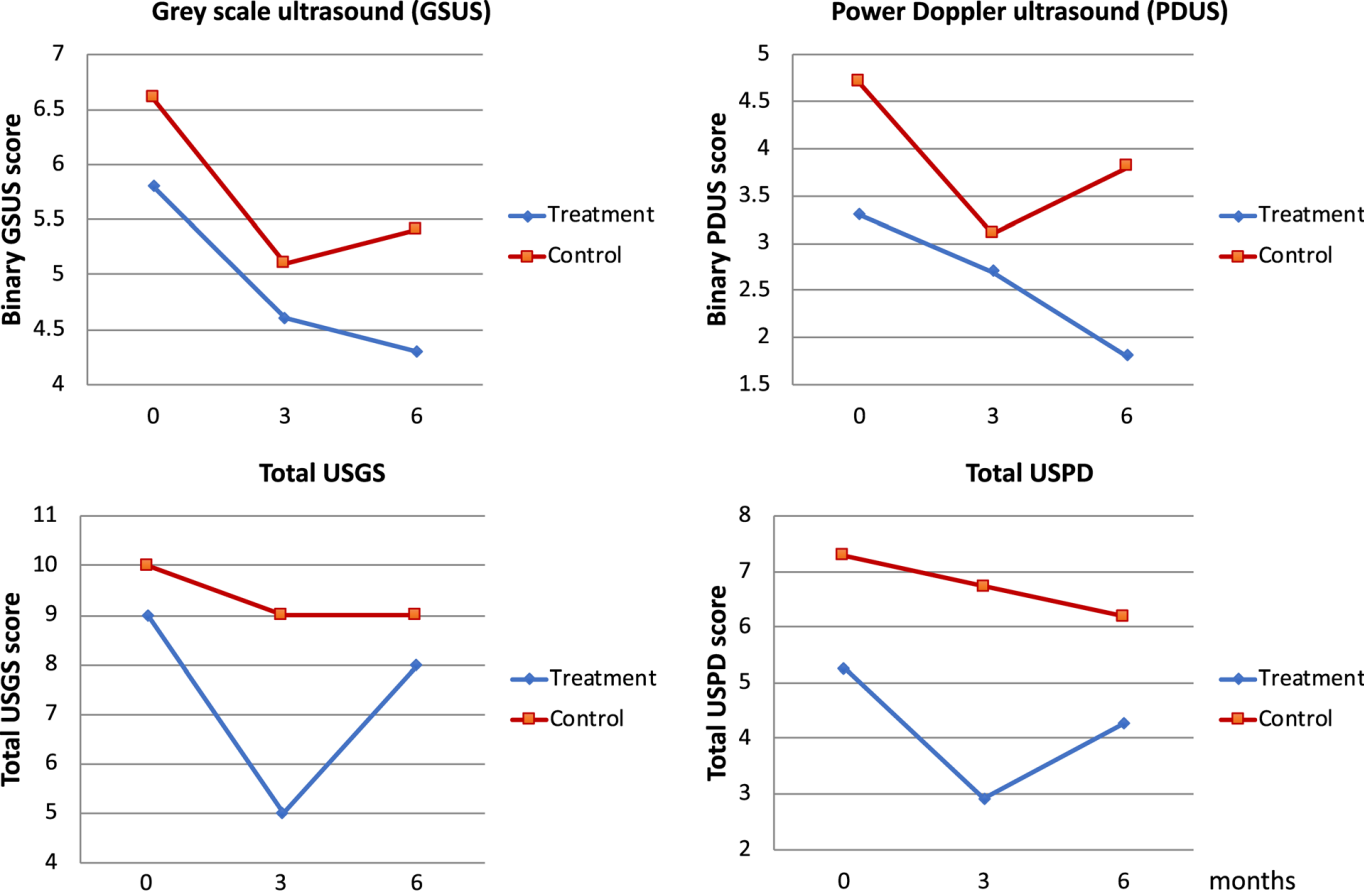
Ultrasound disease activity measures. Ultrasound grey scale (USGS) and power Doppler (USPD) scores were obtained from all metacarpo‐phalangeal (MCP) joints and dorsal wrists on both sides. GSUS, greyscale ultrasound; binary GSUS, sum of greyscale data when converted to a binary variable, with joint involvement based on GS grade of at least 1; total ultrasound greyscale, sum of greyscale grades for all scanned joints; PDUS, power Doppler ultrasound; total USPD, sum of power Doppler grades for all scanned joints.

Both clinical and ultrasound measures of RA disease activity tended to improve in the intervention and control groups. This improvement was more pronounced in the intervention group, particularly over the first 3 months of the trial. There was a higher tender joint count and patient global VAS in the control group, while all of the disease activity components improved in the intervention group (Figure 3). Compared to the control group, the intervention group had improved disease activity scores (DAS28‐CRP) over the course of the study (Figure 4). MSUS scores also improved in the intervention group, particularly Doppler enhancement (Figure 5). There were no differences in the proportion of the medication changes between the groups during the study (17% and 20% in the intervention and control groups, respectively).

## 4 DISCUSSION

We have shown that recruitment into a randomized controlled trial on the effect of periodontal treatment on RA patients is possible and acceptable to participants, although challenging. Out of the total number of patients invited to participate in our study, about a third attended the screening visit, of whom approximately a third were randomized.

More than half of the RA patients invited to participate in the trial declined the invitation for a range of reasons, and a significant number did not attend the screening visit. Although we can only speculate on the reasons for this, a recognized barrier to recruitment and treatment may be related to life experiences such as being employed and/or psycho‐social factors such as dental anxiety (Hill et al., 2013). Indeed, more than 10 million adults suffer from dental anxiety in the United Kingdom, and a 10‐year Office of National Statistics survey of adult dental health published by NHS Digital suggests that over a third (36%) have moderate dental anxiety and as many as 12% of adults may experience high dental anxiety, which results in avoidance of dental care (Hill et al., 2013).

We have also shown that satisfactory retention (i.e., >80%) in such a 6‐month trial is possible, although challenging, for several reasons. Among such factors, RA itself and its comorbidities represent a significant burden, not only in terms of the quality of life but also in terms of logistics (e.g., number of medical appointments and tests needed for this group of patients), which leaves oral health as a health issue of potentially lesser importance compared to other health issues. Indeed, some participants in our study sample received a periodontal assessment and periodontal treatment for the first time through this study despite having long‐standing RA.

The difference in retention between groups at the end of the trial is likely related to the completion of periodontal treatment in the intervention arm, with a resultant lack of interest in attending further study visits. This is a challenge that may lead to selection bias in such trials, and careful consideration needs to be given to incentives for retention.

The study was acceptable to all participants and, using semi‐structured interviews, we have identified several issues to consider that would facilitate retention in future trials (summarized in Serban et al., 2019). The main issues, however, were related to attending several appointments at a site different from the rheumatology clinic site. Indeed, to achieve response, periodontal treatment involves multiple visits in addition to adherence to a regular oral hygiene regime at home.

While periodontal treatment resulted in a reduction in PISA, cumulative PPD, and inflammation markers in our study, the effectiveness of periodontal treatment and patient compliance with an oral hygiene regimen at home should be optimized.

With regard to the eligibility criteria, a small number of volunteers were not judged clinically to have severe periodontitis, although they satisfied the formal inclusion criteria, suggesting that periodontal inclusion criteria should be slightly modified in a definitive study. Some participants met the RA disease activity inclusion criteria with a high patient global VAS score, rather than with a high number of swollen or tender joint counts, raising concerns that in some individuals the disease activity score DAS28 was mainly driven by chronic pain rather than by active disease. Therefore, the inclusion criteria, both in terms of periodontitis and RA disease activity measures, need to be optimized in future studies, including a minimum number of swollen joints and/or imaging with sub‐clinical synovitis as evidence of active inflammation.

The trial duration of 6 months was acceptable to the participants. A study with a longer duration, which would allow the analysis of longer term outcomes, may be difficult to complete for some participants. In addition to contamination, treatment delays in the control group beyond 12 months appear unrealistic in terms of retention. However, based on our patient and public involvement (PPI) discussions, a delay of 6–12 months is acceptable and feasible.

Facilitating study participation is important, including transport issues and communication, and to make it as easy and convenient as possible for participants. In a definitive multi‐centre trial, this will require careful consideration for each site, and the optimal approach may be different for each centre depending upon the local circumstances (e.g., proximity between dental and rheumatology clinics, space, infrastructure at rheumatology clinics, etc.).

Our secondary objective was to collect pilot data regarding the efficacy and safety of intensive periodontal therapy to reduce disease activity for patients with RA and periodontitis. Our data suggest that periodontal therapy resulted in an improvement in RA disease activity measures, including DAS28, inflammation markers, and ultrasound measures. These findings are in line with previous studies, which suggest a beneficial effect of a periodontal intervention in RA disease activity and inflammation biomarkers (summarized in Sun et al., 2021). Given the nature of periodontal treatment, blinding of the intervention was not done; however, we observed improvements in objective measures of both periodontitis and joints based on the MSUS scores obtained, both by blinded examiners. Further larger trials are needed to confirm these results, also including patients at earlier disease phases (e.g., at‐risk populations, clinically suspect arthralgia, unclassified inflammatory arthritis, and early RA).

Based on the key findings of our feasibility study, we suggest the following recommendations to be considered in the design of a future larger trial; however, it is important to note that the feasibility and success of these recommendations would have to be evaluated.

1.Dental screening should be performed in the rheumatology outpatient clinic or clinical research facility (CRF) to maximize capturing potentially eligible patients.

2.Baseline (pre‐randomization) visit should be at appointments separate from the screening visit. This can be at the dental hospital or, ideally, in a fully equipped dental chair near the rheumatology outpatient clinic to facilitate attendance. This also would ensure that participants are willing to attend a dental visit prior to randomization.

3.Having all study assessments (e.g., oral examination, ultrasound) in one place is essential. Ideally, this would be in the rheumatology unit or CRF, as this would expectedly maximize retention, but it has resource implications or may be challenging if recruitment occurs from multiple rheumatology units.

4.Compensation for research visits is strongly advised. Provision of transportation to the research unit for treatment and assessments can be essential depending on local circumstances (particularly if the dental care is in a hospital different from the rheumatology service centre).

5.Provision of other (restorative) dental care is important if patients have dental treatment needs in addition to periodontal treatment. It is recommended not to see a general dental practitioner (GDP) for additional treatment during the study, to improve recruitment and retention and to avoid contamination.

6.The number of treatment appointments should be minimized and, if possible, aligned with routine blood DMARD monitoring tests, provided that such tests are required.

7.A study rheumatology nurse/rheumatologist should be assigned who provides rheumatology care, in alignment with the treating consultant, to ensure protocol adherence and complete data collection with regard to treatment changes and reasons for such changes.

8.A dedicated line of communication should be provided that is easily accessible for all clinical and trial‐related concerns, as well as sending regular reminders to study participants about their upcoming dental appointments. Patients with multiple morbidities often require multiple different hospital appointments and it is important to send timely reminders to reduce the risk of failed appointments.

9.Periodontal treatment at every visit should be provided, and adjunctive use of (local) antibiotics and re‐treatment of all sites should be considered where there is no pocket resolution, with a strong emphasis on oral hygiene instructions and motivation.

10.Provision of an electric toothbrush and other oral hygiene devices, as appropriate, should be considered to enhance compliance with an oral hygiene regimen at home.

11. A sham intervention for the comparator group should be considered in future controlled trials.

12. Ways of strengthening the link between dental and medical care services should be considered as suggested by World Health Organization (2021) and taking advantage of the newly established Integrated Care Systems (ICSs) in England (House of Commons Library, 2021), which present unique opportunities for moving towards greater horizontal service integration delivered by multidisciplinary teams, which include medical, dental, and wider healthcare teams, enabling them to deliver more person‐centred services (Watt & Serban, 2020).

## 5 CONCLUSION

In summary, through this feasibility study we demonstrated that recruitment and retention of RA patients with periodontitis in an RCT comparing immediate to delayed periodontal treatment is challenging but feasible. Satisfactory periodontal treatment outcomes are difficult to achieve in such a trial, and full resolution of periodontal inflammation may not be achievable in some patients. Logistical challenges are significant and require careful consideration, and site‐specific solutions may have to be implemented to keep patients motivated. However, while not powered to assess clinical outcomes, the apparent improvements in RA outcomes in this feasibility study suggest that a definitive trial is worthwhile.

## AUTHOR CONTRIBUTIONS

Paola de Pablo, Stefan Serban, Isabel Lopez‐Oliva, Joanna Rooney, and Thomas Dietrich undertook the project. Paola de Pablo, Iain L. Chapple, Karim Raza, Andrew Filer, and Thomas Dietrich contributed to the conception of the work and interpretation of the findings. Paola de Pablo drafted the manuscript. All authors critically revised the manuscript and approved the final version. The corresponding author attests that all listed authors meet authorship criteria and that no others meeting the criteria have been omitted.

## FUNDING INFORMATION

This work was supported by the National Institute for Health Research (NIHR) under its Research for Patient Benefit (RfPB) Programme (Grant Reference Number PB‐PG‐0609‐19100). Paola de Pablo was supported by an NIHR personal fellowship (Grant Code: NIHR PDF‐2014‐07‐055). Stefan Serban was supported by European Union FP7‐PEOPLE funded ITN project (Project Number: RAPID‐290246). Andrew Filer was supported by Arthritis Research UK (Fellowship 18547). Andrew Filer and Karim Raza were supported by NIHR Birmingham Biomedical Research Centre at the University Hospitals Birmingham (UHB) NHS Foundation Trust and the University of Birmingham (Grant Reference Number BRC‐1215‐20009) and Versus Arthritis Research into Inflammatory Arthritis Centre (RACE) Rheumatoid Arthritis Pathogenesis Centre of Excellence (Grant 20298). The views expressed are those of the authors and not necessarily those of the National Health Service, the NIHR, or the Department of Health and Social Care.

## CONFLICT OF INTEREST

The authors declare no conflicts of interest.

## Data Availability

Research data are not shared.
